# On Seclusion in the Treatment of the Insane
*Report of the Commissioners in Lunacy.


**Published:** 1855-01-01

**Authors:** 


### Art. II.—ON SECLUSION IN THE TREATMENT OF THE INSANE.*

In our last number we gave an analysis of the answers to a circular issued last year by the Commissioners in Lunacy, addressed to the superintendents and medical proprietors of the principal lunatic asylums, and requesting information as to the employment or disuse of instrumental restraint and seclusion in the treatment of the insane, limiting our labours, however, to an elucidation of the practical working of the principle of absolute non-mechanical restraint in the treatment of the insane. Answers were sent by 117 medical attendants or superintendents, 72 of whom were in favour of a qualified use of mechanical restraint, 4 of restraint in surgical cases, 12 gave no opinion, and 29 advocated its total and unqualified abolition, or about 25 per cent. of the whole. The commissioners themselves agree with the large majority of practitioners in not adopting the extreme advocacy of an abstract principle, which the 29 just referred to adopt. They are of opinion "that the possibility of dispensing with mechanical coercion in the management of the insane is, in a vast majority of cases, a mere question of expense"—implying by their phraseology that there *are* exceptional cases—a small minority—to which it is applicable. We may therefore fairly conclude that the controversy as to the unqualified adoption of the abstract principle is now set at rest. Seclusion is substituted, to a large extent, for mechanical restraint, and must necessarily take its place in the controversy also. As to this, we apprehend that the commissioners do but re-echo the unanimous opinion of medical practitioners in stating that its occasional use for short periods, chiefly during paroxysms of epilepsy or violent mania, is generally considered beneficial. They add, however, "that the facilities which seclusion holds out to harsh or indolent attendants for getting rid of and neglecting troublesome patients under violent attacks of mania, instead of taking pains to soothe their irritated feelings, and work off their excitement by exercise and change of scene, render it liable to considerable abuse; and that, as a practice, it is open, though in a minor degree, to nearly the same objections which apply to the more stringent forms of mechanical restraint."

The great majority of the answers mention seclusion as being useful in a few exceptional cases, just as mechanical restraint has been found useful; but there are some who repudiate its use altogether, and some who specially plead in its favour. Dr. Bucknill (who is prominent among the latter) advocates the use of seclusion for two purposes—

* Report of the Commissioners in Lunacy.

first, as a remedial agent; secondly, as a means of coercion. As a remedy, he thinks that it should be made as "agreeable as possible." "The attendants should have the power to enforce seclusion only under the most pressing emergencies, for brief periods, and until the medical officer can arrive. Seclusion being a remedy, should be directed solely by the medical man, whose care it should be to abstract from it every primitive [?] characteristic. The easiest mode of doing this is to invest it with a medical character; to speak of it as necessary for health, and even to add some other remedy more purely medical." Dr. Bucknill has noticed *accidental* seclusion in bed (as for a sore leg) to be beneficial, and in a few cases this plan might be adopted; otherwise he recommends that it be practised either in the open air, in an airing court, or in light and cheerful sitting-rooms, furnished with the means of occupation and amusement.

"There is, however," Dr. Bucknill remarks, "another aspect under which seclusion must be considered, wherein it is not remedial, wherein it is acknowledged to be an evil, by its use being justified as the least of two evils, of which the annoyance and danger of the patients in general is the greater. It cannot be denied that insanity frequently displays itself by excitement of the malignant passions; and that some of the most depraved of mankind terminate their career in asylums. Towards these seclusion must occasionally be employed in its harsher form as a coercive means, to prevent the welfare of the many from being sacrificed to the passions of the few." In other words, mischievous and malignant inmates must be *placed in solitary confinement*—emphatically, in "seclusion in its harsher form."

Dr. Diamond, of the female division of the Surrey County Asylum, wholly repudiates the use of seclusion, and scatters all Dr. Bucknill's euphonisms and nice distinctions by the results of his experience. "Seclusion," he observes, "or solitary confinement of patients in a separate room against their will, I also much object to. I have no doubt cases may occur in which this may be requisite and beneficial, but they must be of rare occurrence. *I have not had a single patient* under seclusion during the past twelve months; and during the year 1852 it was used only in two instances for a period of nineteen hours in the whole. The discontinuance of seclusion has produced the greatest possible good; and I appeal to all who have visited the wards of this asylum to speak to the great quietness and industry which prevail throughout. I have now under my care patients who broke windows and committed all sorts of violence in order to be placed in seclusion, where they might rest in idleness, wrapped up with a rug in a corner of a cell for hours together, but who are now industrious persons, although their mental state is the same."

Now let us contrast this disuse of seclusion by Dr. Diamond with its use by others. First, as to Dr. Bucknill, its special advocate. "During the past year (1853)," he writes, "the total number of seclusions of female patients in the Devon Asylum has been 164, or rather more than an average of three a week. The average duration of all these instances of seclusion added together was eighteen hours and three minutes in each week. The average duration of each instance of seclusion was five hours and forty-four minutes. The average number of female patients was 260. The total number of male patients during the past year was 58, or rather more than an average of one in a week. The average duration of these seclusions was nine hours and twenty-five minutes in each week; and the average duration of each instance of seclusion was eight hours and twenty-five minutes. The average number of male patients was 200." It will be observed that the number of female patients under Dr. Diamond's care is just double that under Dr. Bucknill's.

Supposing, therefore, Dr. Bucknill had been placed in Dr. Diamond's place, his use of seclusion with the greater number would, as compared with that of the latter, be as follows:—

Dr. Bucknill's seclusions per annum ... ... ... 328
Dr. Diamond's seclusions per annum ... ... ... 0
Or, in time, Dr. Bucknill secluded for 11 weeks in 1853.
" " Dr. Diamond—not for an hour!

But if we take the two years, 1852, 1853, we might almost double these figures again.

These are enormous practical differences in the two methods of treatment; but are they so inexplicable as to warrant Dr. Diamond in asserting, that any person who would now use seclusion to the extent Dr. Bucknill has used it is unfit to have the superintendence of an asylum? Most decidedly not: for we do not believe that either mechanical restraint or seclusion has been so entirely disused in the Surrey Asylum as Dr. Diamond fondly believes. In venturing this assertion we do not intend or desire in the least degree to impugn that gentleman's veracity; we are satisfied that his statements are all made *bonâ fide.* We will state our reasons for our opinion. Finally, we have a large amount of evidence from perfectly trustworthy sources (Dr. Bucknill, for example) as to the *necessity* of seclusion in, at least, a few cases. Examples we subjoin.

Dr. Thurnam, of the Wilts County Asylum, remarks:—"The proportion of cases in which seclusion is resorted to is very small. Out of an average of 250 patients of both sexes, it is rare to have two cases under seclusion at the same time, or to have more than one or two instances during the week, and these *generally confined* to the *female* department."

Mr. Stevens, of St. Luke's, writes: "The amount of seclusion has also been very trifling during the past year, averaging two cases per week, and these for periods of a few hours only at a time. The patients so treated having been, from indecency of conduct, violence to others, or general turbulence of behaviour, entirely unfitted, for the time, for association with the other inmates."

So also Mr. Allen, of the Darneford Asylum: He secludes for mischievous destruction of everything within reach, extreme violence and assault, incessant shouting, blasphemous swearing, indecent and disgusting language—cases in which seclusion is used to attain Dr. Bucknill's second object—namely, *coercion* of the violent and depraved.

We could multiply quotations; but these suffice for our purpose, which is to show a concurrence with Dr. Bucknill, so far as this—that seclusion of a lunatic is occasionally absolutely necessary for the peace and comfort of the other inmates.

But, secondly, we do not believe that either mechanical restraint or seclusion has been so *entirely* disused in the Surrey County Asylum as Dr. Diamond states, for another reason—namely, that there is no evidence in support of the assertion. That gentleman, we venture to believe, eats and sleeps, and takes his recreation, like other superintendents; he is not, therefore, *always* in the wards of his establishment, nor is he, being mortal, ubiquitous. Yet, to gain even a hearing for his assertion that, during five years, in "not a single instance" of 800 cases under his care "has *any* restraint been used," he must first show that he was in *every* ward at *every* moment of the twenty-four hours for two entire years! Otherwise his statement is only made on hearsay evidence. Now, hearsay evidence is not admissible, except with much corroboration; hearsay *negative* evidence is inadmissible altogether.

Dr. Conolly has had too much experience of the tricks of attendants in asylums to believe all that he is told, or to venture on such an unqualified and wholly inadmissible assertion as Dr. Diamond has ventured upon.

Dr. Conolly wisely qualifies his evidence as to Hanwell thus: "No form of mechanical restraint was employed, *with my knowledge or sanction*, by night or by day;" but that it was used without his knowledge and sanction, and is often used in asylums, especially the overgrown county establishments, without the knowledge of the officers, is deducible enough from the following description of the supervision, and the conduct and character of the attendants of large asylums:—

Dr. Conolly states—"The supervision of the attendants in the *large* asylums is *almost always* inefficient. The female attendants do not often remain long enough in them to learn their duties; and in some of them they only learn to avoid trouble, by having recourse to mechanical restraints in every difficulty. The male attendants usually retain their situations longer; but in consequence of the duties of a large asylum being generally too great in proportion to the medical staff, they know themselves to be for a considerable portion of the day free from observation; and they learn to baffle even the inspection to which they are subjected, by signals and other acts of confederacy, and in some cases establish an organized ruffianism which long escapes detection, and which some frightful outrage only at length reveals."

Dr. Diamond will do well to ponder these statements, and perhaps, when his perceptions have been quickened a little, he will discover that the females' division of the Surrey County Asylum is not the paradise he fondly imagines it to be.

A more important consideration remains, namely—that Dr. Diamond is wrong in principle—wholly wrong. Insanity comprises a group of diseases of the nervous system, more particularly of that portion in relation with sensorial stimuli. All medical experience as well as medical philosophy teaches that when these stimuli excite still more a morbidly excited and unduly active sensorium, they should be prevented reaching it as far as practicable. Hence the obvious and generally acknowledged advantage of seclusion in encephalic inflammations, in acute mania, in some forms of epilepsy, and in certain morbid transitory states of the nervous system, during which everything is a source of discomfort or irritation. We think it altogether incredible that amongst 520 female patients not a single instance of acute cerebral inflammation or of acute mania or of excessive irritability of the sensorial centres occurred during a whole twelvemonth, and it appears to us almost equally incredible that in such a case Dr. Diamond has not directed the best approved, most simple, and most effectual means of alleviation, namely seclusion. We will go further, and say that we are confident Dr. Diamond has often used this remedial agent, but under another name. In short, our conclusion is that both as to the use of mechanical restraint and seclusion the only difference between Dr. Diamond and others is as to the meaning of terms. This we gather, certainly, as to seclusion, from Dr. Diamond's use of the word "*cell*" in reference to it,—an obsolete word, that ought to be wholly banished from the literature of modern psychiatry.

But the question arises, whether the seclusion practised by Dr. Bucknill was wholly necessary. It *seems* to have been excessive, but the excess may be only seeming. If a superintendent administer morphia, or any other preparation of opium largely, he may boast of his rare cases of mechanical restraint and seclusion, but he ought not to boast of his skill and judgment in the treatment. Or he may cure a refractory patient by the use of the cold bath or shower-bath, and fairly boast that he never uses *mechanical* restraint, or seclusion; but if he pharisaically claim to be superior in benevolence and humanity to those who do use them when they think best for the patient, we have no hesitation in saying that the charge of inhumanity and cruelty rather rests upon him.

The treatment of the insane should differ as little as possible from that of the sane, when they are under *similar* circumstances of health or disease. If an opiate or other medicinal sedative be indicated, by all means let it be administered; if the shower-bath be thought advisable, use it; if isolation from stimuli be beneficial, place the patient, without hesitation, in seclusion; if deliriously moving hands and feet are mischievous, do not hesitate in restraining them by any benevolently appropriate means; but above all let not the medical practitioner, from any foolish fear of incurring censure or obloquy, restrain his own freedom of action, or the freedom of action of his brethren, by the dogmatic and intolerant enunciation of abstract principles. Such conduct is subversive of all independent manly thought, and will inevitably bring on those who adopt it the imputation of cant, humbug, bigotry—things discreditable to the noble art of physic.

---

